# Changes in hippocampal volume, synaptic plasticity and amylin sensitivity in an animal model of type 2 diabetes are associated with increased vulnerability to amyloid-beta in advancing age

**DOI:** 10.3389/fnagi.2024.1373477

**Published:** 2024-06-21

**Authors:** Melih Tarhan, Tim Hartl, Olena Shchyglo, Jens Colitti-Klausnitzer, Angela Kuhla, Tobias Maximilian Breuer, Denise Manahan-Vaughan

**Affiliations:** ^1^Department of Neurophysiology, Institute of Physiology, Ruhr University Bochum, Bochum, Germany; ^2^International Graduate School of Neuroscience, Bochum, Germany; ^3^Rudolf Zenker Institute for Experimental Surgery, Rostock University Medical Center, Rostock, Germany

**Keywords:** amyloid-beta, amylin, MRI, diabetes, hippocampus, rodent, synaptic plasticity, patch clamp

## Abstract

Type-2 diabetes (T2D) is a metabolic disorder that is considered a risk factor for Alzheimer's disease (AD). Cognitive impairment can arise due to hypoglycemia associated with T2D, and hyperamylinemia associated with insulin resistance can enhance AD pathology. We explored whether changes occur in the hippocampus in aging (6–12 months old) female V-Lep^○b^-/- transgenic (tg) mice, comprising an animal model of T2D. We also investigated whether an increase in vulnerability to Aβ (1–42), a known pathological hallmark of AD, is evident. Using magnetic resonance imaging we detected significant decreases in hippocampal brain volume in female tg-mice compared to wild-type (wt) littermates. Long-term potentiation (LTP) was impaired in tg compared to wt mice. Treatment of the hippocampus with Aβ (1–42) elicited a stronger debilitation of LTP in tg compared to wt mice. Treatment with an amylin antagonist (AC187) significantly enhanced LTP in wt and tg mice, and rescued LTP in Aβ (1–42)-treated tg mice. Taken together our data indicate that a T2D-like state results in an increased vulnerability of the hippocampus to the debilitating effects of Aβ (1–42) and that effects are mediated in part by changes in amylin receptor signaling.

## 1 Introduction

Type 2 diabetes (T2D) is a widespread metabolic disorder, which affects more than 415 million people worldwide (Chatterjee et al., 2017). It causes many severe complications, not only affecting the cardiovascular and peripheral nervous system, but also the central nervous system (Biessels et al., 2014) T2D is also considered a risk factor for Alzheimer's disease (AD; Biessels et al., 2002; Kopf and Frölich, 2009; Pruzin et al., 2018). AD is a neurodegenerative disorder and the most common form of dementia. In 2019, it was estimated that over 47 million people worldwide suffer from dementia and that this number will increase 3-fold million by 2050 (Tiwari et al., 2019). Roughly 70% of dementia cases are attributed to AD (Reitz et al., 2011). Cerebral plaques consisting of β-amyloid peptide (Aβ) are one of the important pathological hallmarks of Alzheimer's disease (Querfurth and LaFerla, 2011; Selkoe and Hardy, 2016) and Aβ(1–42) has been identified as a key factor in its pathogenesis (Younkin, 1998; Mroczko et al., 2018).

A prominent feature of T2D is leptin dysfunction comprising leptin-resistance and leptin-signaling deficiency (Licinio et al., 2004; Zhou and Rui, 2013; Salazar et al., 2020). Leptin is a 16 kD adipokine-polypeptide hormone (Zhang et al., 1997). It comprises 167 amino acids that are encoded by the obesity (ob) gene (Zhang et al., 1994; Tartaglia et al., 1995). The interconnection of leptin with T2D has been proposed to arise because obese patients develop abnormally high levels of leptin, leading to leptin-resistance (Knight et al., 2010). This process in turn, results in a failure of leptin to reduce appetite and thus, progressive weight-gain is further propagated (Zhou and Rui, 2013). In transgenically modified rodents, leptin deficiency, or a lack of a functional leptin receptors, leads to morbid obesity and T2D (Zhang et al., 1994; Yin et al., 2017). Treatment of T2D patients using leptin replacement has been reported to improve their metabolic disorder status, to reduce insulin insensitivity and reverses obesity (Farooqi et al., 2002; Gibson et al., 2004; Licinio et al., 2004; Paz-Filho et al., 2008), suggesting that leptin dysfunction may also directly affect glucose metabolism (Margetic et al., 2002). Furthermore, deficient leptin signaling results in changes in brain neurovascular structure, and increased activation of microglia in cortex and hippocampus in transgenic animal models of both T2D and obesity (Hayden and Banks, 2021), thereby suggesting that leptin may also mediate these disorders by changing cognitive information processing. Substantial evidence exists that leptin regulates hippocampal function and thus, hippocampus-dependent learning and memory processes (McGregor and Harvey, 2018). More recently, it has emerged that leptin deficiency is a risk factor for AD (Flores-Cordero et al., 2022).

In animal models of AD and T2D, impairments of hippocampal synaptic plasticity (Kalweit et al., 2015; Sasaki-Hamada et al., 2015; Yin et al., 2017) and spatial learning (Kalweit et al., 2015) have been reported. Both disorders lead to brain atrophy (van Harten et al., 2006; Reijmer et al., 2010). Although it is believed that a relationship exists between AD and T2D (de la Monte and Wands, 2008; Crane et al., 2013) the exact link is not clear (Yang and Song, 2013). Here, one possible candidate comprises amylin. It is a 4 kDa pancreatic peptide that is co-released with insulin that supports energy metabolism, satiety and neuronal development (Hay et al., 2015; Levin and Lutz, 2017; Zhang and Song, 2017; Flores-Cordero et al., 2022). Its primary site of action in the brain is the area postrema, and action of amylin at this site decreases food intake by prompting the ending of meal engagement (Riediger et al., 2004). Interestingly, amylin restores leptin sensitivity in obese humans and rodents (Roth et al., 2006; Trevaskis et al., 2008). Furthermore, mice that lack leptin receptors exhibit a reduced appetite-suppression response to amylin (Duffy et al., 2018). Amylin and leptin depolarise the neuronal population in the area postrema, suggesting that co-activation by amylin and leptin is necessary for appropriate appetite regulation (Smith et al., 2016). In T2D, amylin becomes dysregulated and leads to the formation of pancreatic islet amyloid through self-association (Cooper et al., 1987; Westermark et al., 1987; Mukherjee et al., 2015; Akter et al., 2016). Amylin may also play an important role at the interface between T2D and AD (Lutz and Meyer, 2015). It exacerbates the effects of Aβ(1–42) in the brain and promotes plaque formation (Andreetto et al., 2010). Furthermore, amylin receptor expression is increased in association with amyloid burden (Jhamandas et al., 2011). In T2D patients, hyperamylinemia is common (Despa et al., 2012) suggesting a putative link between changes in amylin receptor function in T2D and the vulnerability of these patients to AD.

In this study, we explored to what extent the hippocampus is altered in middle-aged (6–12 month old) transgenic mice that lack functional leptin receptors and become morbidly obese in adulthood (V-Lep^○b^-/- mice), corresponding to an animal model of T2D (Wasim et al., 2016; Kleinert et al., 2018). Our first goal was to explore to what extent a T2D-like state may lead to changes in hippocampal volume, or synaptic plasticity that could forbode a putative vulnerability to Aβ(1–42). We previously reported that treatment of the hippocampus with oligomeric Aβ(1–42) impairs long-term potentiation (Kalweit et al., 2015). Given the connection between leptin-deficiency and AD (Flores-Cordero et al., 2022), our second goal was to explore to what extent the debilitation of LTP by oligomeric Aβ(1–42) differs in healthy and V-Lep^○b^-/- mice. Genetic depletion of amylin receptors improves learning and memory in AD mouse models (Patel et al., 2021). Thus, our third goal was to investigate to what extent amylin antagonism affects hippocampal LTP in the presence or absence of Aβ(1–42). Finally, given the putative interconnection between amylin and leptin in regulating appetite and brain function, our fourth goal was therefore, to clarify whether differences in the sensitivity of the hippocampus to amylin occur in V-Lep^○b^-/- mice and whether amylin receptor antagonism can ameliorate the effects of Aβ(1–42) on hippocampal LTP in V-Lep^○b^-/- mice.

## 2 Materials and methods

### 2.1 Animals and ethical permission

The study was carried out in accordance with the European Communities Council Directive of September 22nd 2010 (2010/63/EEC) for care of laboratory animals and after approval of the local government authority [Landesamt für Arbeitsschutz, Naturschutz, Umweltschutz und Verbraucherschutz (LANUV), Arnsberg, Germany]. The experimental protocol was registered and approved in advance with LANUV. All efforts were made to minimize the number of animals used. Age matched 6–12 months old female B6.Cg-Lep^○b^/J (B6. V-Lep^○b^-/-, obese, homozygote) mice (JAX stock #000632, Zhang et al., 1994) and their female lean (B6. V-Lep^○b^-/+, heterozygote), or wild-type, littermates (B6. V-Lep^○b^+/+) were used (Jackson Laboratory, Bar Harbor, ME, USA) in accordance with §4 (German animal welfare act, version from 2017). Mice were housed in a temperature- and humidity-controlled vivarium (Scantainer, Scanbur, Denmark) with a constant 12-h light-dark cycle (lights on from 6 a.m. to 6 p.m.) with *ad libitum* food (ssniff^®^ R/M-H, ssniff Spezialdiäten GmbH, Soest, Germany) and water access. Food and water supplies were replenished on a daily basis. Humidity and temperature in the containers were maintained at ca. 20°C and ca. 50%, respectively. The vivarium minimizes sound entry so that the animals were not subjected to unexpected sound stress. Mice were housed in littermate groups in macrolon containers (Eurostandard Type III (elevated), 425 mm long × 266 mm width × 185 mm height), with metal grid lids that contained a depression for food access and for the water bottle nozzle. These “homecages” were placed side-by-side in the housing containers, so that mice could see, smell and hear their neighbors (Manahan-Vaughan, 2018). To reduce the risk of social stress and allow for species-appropriate group housing, only females were used in the study.

For *in vitro* electrophysiology, animals were quietly removed from the vivarium, their home cages were covered with a thick cotton sheet and they moved to a nearby lab to commence procedures. For magnetic resonance imaging experiments, animals were transported to the animal housing unit of the imaging facility at least 1 day before experimentation. The housing room is immediately adjacent to the scanner unit and preparation room, and animals were transported quietly in their covered homecages to the preparation room prior to commencing procedures.

Homozygous mutant mice develop a spontaneous mutation of leptin and become obese at about 4 weeks of age, gaining roughly three times the normal weight of wild-type controls by 16 weeks of age (see information provided by the Jackson Laboratory website: www.jax.org/strain/000632). Transgenic mice were carefully monitored to ensure that they did not “capsize” due to their rotund form and were placed back on their paws if this occurred. Humane endpoints such as changes in external physical appearance, behavior, clarity of the eyes, were monitored on a daily basis. The health status of animals was additionally assessed weekly by the veterinarian of the Medical Faculty of Ruhr University Bochum.

### 2.2 Genotyping of transgenic mice

Genotyping was conducted according to the Jackson Laboratory protocol (www.jax.org/strain/000632). Briefly, for DNA preparation, ear punches were taken from <3 week old mice and were incubated for 1 h at 97°C in 50 mM NaOH in 1.5 ml sealing tubes. After cooling, 1M Tris/HCl (pH = 8) was added to the well-mixed sample, centrifuged at 10,000 × *g* for 1 min, and stored at −20°C until genotyping by means of PCR. The PCR protocol (no. 551 “Restriction Enzyme Digest Assay”) was followed as described by the Jackson Laboratory (www.jax.org/strain/000632). Here, Reaction A (with the components: 10x PCR Buffer (Invitrogen, CA, USA), 25 mM MgCl_2_ (Invitrogen), 10 mM dNTP (Invitrogen), 20 μM oIMR1151 (fwd: 5'-TGT CCA AGA TGG ACC AGA CTC-3'), 20 μM oIMR1152 (rev: 5'- ACT GGT CTG AGG CAG GGA GCA-3'; both from eurofins, Ebersberg, Germany), and 5 U/μl Taq polymerase (Invitrogen) were run with the following PCR cycling steps: **1**. 94°C for 3 min, **2**. 94°C for 30 s, **3**. 62°C for 1 min, **4**. 72°C for 45 s (repeat steps 2–4 for 35 cycle) and 72°C for 2 min. In addition, Reaction B (digestion step with components: 10x buffer (cut smart), 10 mg/ml BSA and Dde1 (all from New England BioLabs, Frankfurt a.M., Germany) was added to the PCR products and incubated for 37°C for 6 h. Afterwards, DNA loading dye (Peqlab Biotechnology, Erlangen, Germany), was added to digested PCR products and separated on a 3% agarose gel. The following PCR products were detectable: mutant (Lep^○b^-/-) = 55 and 100 bp, heterozygote (Lep^○b^-/+) = 55, 100, and 155 bp, and wt (Lep^○b^+/+) = 155 bp.

### 2.3 Magnetic resonance imaging

Before measurements were commenced, animals were anesthetized with isoflurane in O_2_:N_2_O. Mouse brains were assessed by means of 7-Tesla magnetic resonance imaging (MRI; Bruker Avance Biospec 70/30 USR, Karlsruhe, Germany) using a vendor transmit receive quadrature mouse cryogenic coil and its dedicated mouse bed. The body temperature was maintained around 37.5°C and was continuously monitored (Model 1025T, Small Animal Instruments Inc., New York, NY, United States).

**Figure 1:**
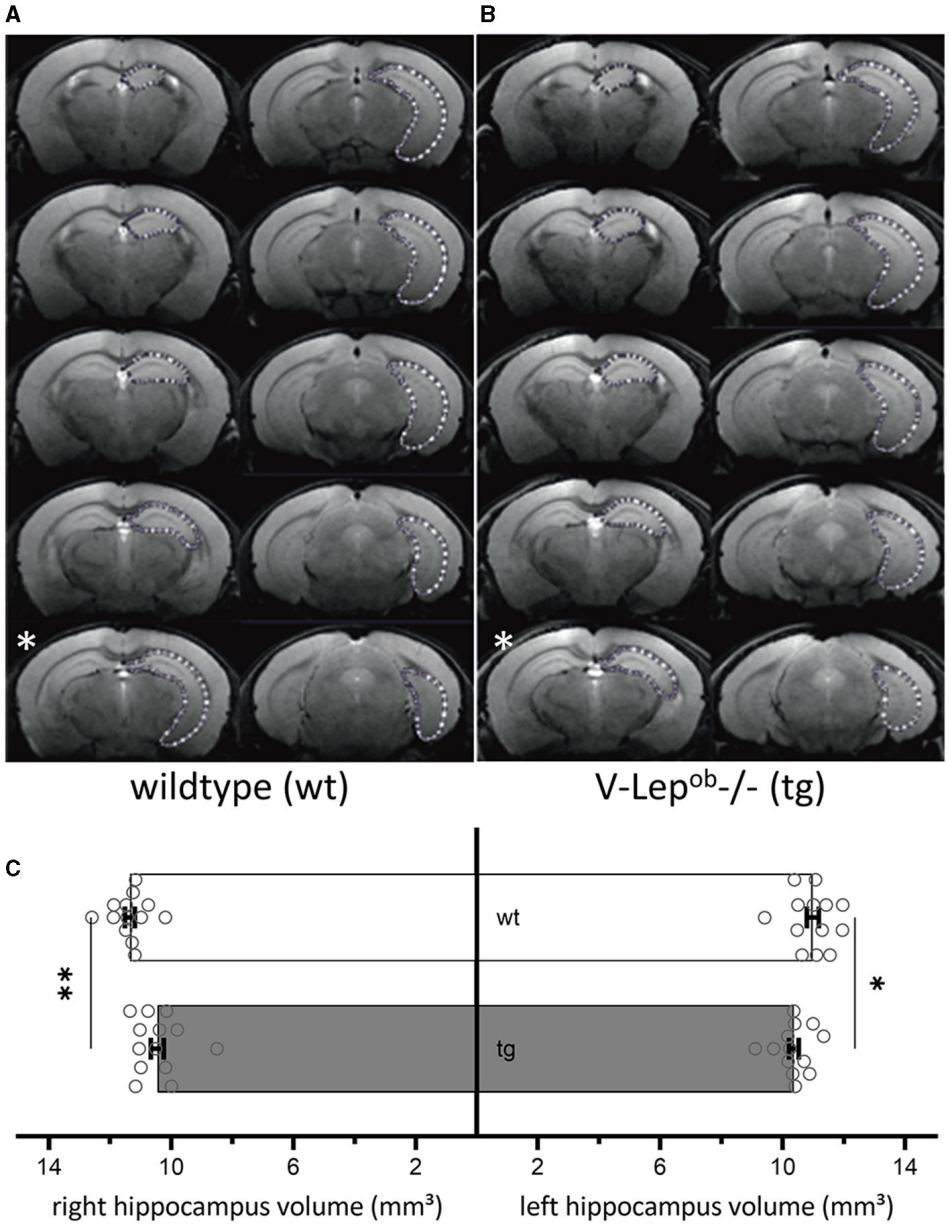
Volumes of bilateral hippocampi are reduced in V-Lep^○b^-/- (tg) animals compared to wildtype littermates. **(A, B)** Series of coronal MR images of a wildtype [wt, **(A)**; *n* = 13, *N* = 9] and a transgenic (tg) mouse **(B)** (*n* = 13, *N* = 9) showing the manual outlining of the left hippocampus for MR volumetry (white dotted lines). The white asterisk (bottom left of each panel) marks a possible acquisition irregularity in a slice that was excluded from analysis from each respective cohort. **(C)** The volume of both the right and the left hippocampus of tg mice is significantly smaller compared to wildtype hippocampi; two-way ANOVA: Genotype: *F*_(1,48)_ = 17.19, *p* = 0.0001, hemisphere: *F*_(1,48)_ = 0.1.54, *p* = 0.22; *post-hoc* Fisher's LSD: wt-rightHC vs. tg-rightHC: *p* = 0.022; wt-leftHC vs. tg-leftHC: *p* = 0.001). No differences were evident between hemispheres within genotypes (Fisher's LSD: wt-rightHC vs. wt-leftHC: *p* = 0.16; tg-rightHC vs. tg-leftHC: *p* = 0.76). Data are given as mean ± SEM (as error bars). Significant levels: *p < 0.05, **p < 0.01.

Animal orientation in the scanner was carefully assessed for optimal reproducibility by serial recordings and the angular adjustment of three (first axial, then horizontal, and finally sagittal) pilot rapid-acquisition relaxation-enhancement (RARE) 2D images. The horizontal images were kept orthogonal to the axial images, the sagittal images were kept orthogonal to the horizontal images. Then 44 coronal high resolution 2D RARE images were acquired with an activated fat saturation module (repetition time 5,500.8 ms, effective echo time 45.0 ms, RARE factor 4, matrix size 256 × 256, field-of-view 18 × 18 mm^2^, resolution: 0.070 × 0.070 × 0.3 mm^3^, two averages, total acquisition time: 11 min, 44 s). These images were binned into 10 optical slices per animal, representing a depth of 3 mm along the ideal line between the olfactory bulb's superior end and the posterior end of the cerebellum. The binned optical slices were then aligned starting from the most posterior (Figures 1A, B).

### 2.4 Hippocampal slice preparation

For electrophysiology, 400 μm hippocampal slices were prepared in cold (1–4°C), oxygenated artificial cerebrospinal fluid (aCSF; in mM: 124 NaCl; 4.9 KCl; 1.2 NaH_2_PO_4_; 1.3 MgSO_4_; 2.5 CaCl_2_; 25.6 NaHCO_3_; and 10 D-glucose; pH 7.4) Slices were continuously perfused at a constant flow rate of 2 ml/min with oxygenated aCSF at 30°C prior to electrophysiological recordings, to allow the slices to recover from the mechanical stress of the dissection and to equilibrate to the recording temperature.

### 2.5 fEPSP recordings

For stimulation, a bipolar stimulating electrode (Fredrick Haer, Bowdowinham, ME, USA) was placed in the Schaffer collaterals of the hippocampus and a glass recording electrode (impedance: 1–2 MOhm) filled with aCSF was positioned in the ipsilateral CA1 stratum radiatum. Test-pulse stimuli of 0.2 ms duration at a frequency of 0.025 Hz were applied, and evoked field excitatory post-synaptic potentials (fEPSPs) were recorded with a sample rate of 10,000 Hz. For each time point, five responses were averaged. Before recordings were started, a stimulus-response relationship was determined using a stimulation range of 50–600 μA in 50 μA steps, applied every 60 s, to detect the maximal fEPSP. The stimulation strength used for subsequent test-pulses comprised the stimulus intensity that evoked ~50% of the maximal fEPSP. After baseline recordings for 40 min, LTP was induced by using high frequency stimulation (HFS) with three trains of 100 pulses at 100 Hz delivered at 5 min intervals. An overview of the number of animals and slices used for these experiments is provided in Table 1.

**Table 1:** Overview of animal group numbers and slices for field potential (fEPSP) experiments.

**fEPSP recordings: overview of animal numbers and slices (** **Figure 2** **)**			
**Type of experiment/ treatment**	**Genotype**	**Animals**	**Slices**
HFS, membrane excitability (data not shown)	wt	12	16
	tg	10	23
LTP control (Figure 2A)	wt	5	6
	tg	5	8
LTP: Aβ (Figure 2B)	wt	5	8
	tg	5	6
LTP: AC187 (Figure 2C)	wt	8	8
	tg	5	8
LTP: Aβ + AC187 (Figure 2D)	wt	5	6
	tg	5	7

The table offers an overview of the number of animals and slices used in fEPSP experiments, for wild-type (wt) and transgenic (tg) mice. In the left column, the figure, in which these data are graphically reported, is mentioned.

### 2.6 Patch clamp recordings

Patch clamp recordings were conducted as described previously (Novkovic et al., 2015), using brain slices from the same animals used for the abovementioned LTP experiments. Hippocampal slices were maintained at room temperature for 30 min before transfer to a recording chamber that was located on an upright microscope. Slices were continuously perfused with oxygenated aCSF (at a constant flow rate of 1–1.5 ml/min). Recording pipettes were pulled from borosilicate glass pipettes (1.5 mm external diameter) with a resistance of 6–14 MOhm and were then filled with intracellular solution (in mM: 97.5 potassium gluconate, 32.5 KCl, 5 EGTA, 10 Hepes, 1 MgCl_2_, 4 Na_2_ ATP, adjusted to pH 7.3 with KOH). Recordings were performed from visually determined cell bodies of pyramidal neurons in the CA1 somatic region.

Intrinsic membrane properties were acquired with a HEKA EPC10 amplifier using the PATCHMASTER acquisition software (HEKA). Data were subjected to low-pass filtering at 2.9 kHz and digitized at 10 kHz. FITMASTER (HEKA) and APfeature (Matlab computer runtime) were used for offline analysis. Input resistance was deduced from the slope of the linear fit of the relationship between the change in membrane potential (ΔV) and the intensity of the injected current (between −120 and +90 pA). The time constant was determined from an exponential fit of the averaged voltage decay. The mean of 30 s basal recording time was taken as the resting membrane potential. The minimum current needed to induce an action potential was taken as a threshold current. The action potential amplitude was measured as the voltage difference between the threshold and the peak. Firing properties were examined by applying current steps of Δ50 pA hyperpolarizing and depolarizing square pulses (1-s duration) through the patch-clamp electrode (in the range of −300 to 400 pA).

Aβ(1–42) was applied using a closed circuit of aCSF (60 ml) containing 500 nM Aβ (1–42; Wang et al., 2004). Slices were treated for 10 min with Aβ(1–42) before recordings commenced. An overview of the number of animals and slices used for these experiments is provided in Table 2.

**Table 2:** Overview of animal group numbers and slices for patch clamp experiments.

**Patch clamp recordings: overview of animal numbers and slices (** **Figure 3** **)**			
**Treatment**	**Genotype**	**Animals**	**Cells**
Control	wt	8	22
	tg	12	22
Aβ	wt	5	19
	tg	5	20
AC187	wt	5	25
	tg	5	26
Aβ + AC187	wt	6	23
	tg	5	22

The table offers an overview of the number of animals and slices used in patch clamp experiments, for wild-type (wt) and transgenic (tg) mice under different treatment conditions.

### 2.7 Amyloid-beta preparation

The soluble Aβ (1–42) peptide was prepared in PBS at p.H. 7.4, as previously described (Kalweit et al., 2015). It was subsequently diluted to the concentration of 50 μM, shock-frozen with liquid nitrogen and stored at −80°C. The Aβ solution was incubated for 3 h one day before the experiment to ensure that oligomerization occurred (Kalweit et al., 2015) and subsequently stored at −80°C. It was thawed at room temperature 5 min before application to the slice preparation. The Aβ solution was added to the aCSF so that the final concentration during the experiments was 500 nM (Ondrejcak et al., 2012) and applied directly to the hippocampal slice via bath perfusion.

### 2.8 AC187 treatment

AC187 pharmacologically antagonizes the neuronal amylin receptor (Jhamandas and MacTavish, 2004). It has been reported to prevent Aβ-mediated toxicity in the basal forebrain (Jhamandas and MacTavish, 2004). In our studies, AC187 (Abcam plc, Cambridge, U.K.) was diluted in water and directly applied to the hippocampal slices via bath perfusion in a concentration of 250 nM. After 30 min of baseline recordings, the application of AC187 began. Application was continued in a closed circuit until the end of the experiments.

### 2.9 Data analysis and statistics

#### 2.9.1 MRI data

To calculate hippocampal volume, the area of the hippocampus of 13 animals per genotype was determined on each slice containing the hippocampus. For this purpose, regions of interest (ROIs) were drawn manually with close reference to a mouse brain atlas (Paxinos and Franklin, 2012) using the program ImageJ (Figures 1A, B). Due to inequal orientation of anatomical landmarks in some optical slices, the affected slice was selectively excluded from each animal, therefore only 9 slices were used for the volumetry (see slice 5 in Figure 1B, marked with a white asterisk). In total 13 animals were used of which nine optical slices were analyzed after excluding slice that contained artifacts that precluded data analysis. The volume of hippocampus was determined by the sum of the area of the ROI from the analyzed slices multiplied by the slice thickness. Results were analyzed as one group with two factors: genotype and hemisphere. After confirmation of normal distribution and variance homogeneity (Shapiro-Wilk Test), one-way analysis of variance (ANOVA) was applied. Multiple comparisons between each group were done using an uncorrected Fisher's LSD test, *post-hoc*.

All data were shown as mean ± standard error of mean. For all statistical results, the level of significance was set at *p* < 0.05 (animals *n* =13, slices *N* = 9).

#### 2.9.2 Electrophysiological data

LTP responses between the groups were analyzed by repeated measures ANOVA and *post-hoc* Fisher's LSD Test, following the confirmation that the data were normally distributed (Shapiro-Wilk Test). Mean ± the standard error of the mean (s.e.m.) are reported. For the statistical analysis of LTP experiments, fEPSPs evoked from 5 min post-HFS onwards were assessed (i.e., *t* = +5 min through *t* = +60 min post-HFS).

Following confirmation of normal distribution, a comparison of stimulus-response relationships (fEPSP slope) was conducted using an unpaired Student's *t*-test. In addition, a non-linear fit model was calculated to be used as a trend line.

To assess changes in LTP responses, data obtained at the 60 min timepoint post-HFS were compared. One single outlier was determined (ROUT-Method Q = 1%). Finally, a two-way ANOVA was applied to test for the factors genotype, treatment and interaction. Subsequent multiple comparisons were done via the uncorrected Fisher's LSD test.

Since there are two genotypes and four possible treatments the statistical analysis of passive and active membrane properties was done using a two-way ANOVA. All comparisons were followed by an uncorrected Fisher's LSD test for multiple comparisons. All data sets underwent outlier analysis (ROUT-Method Q = 1%) before comparison.

For LTP experiments, “n” corresponds to the number of animals and “N” corresponds to the number of slices in the experiment. For patch clamp data, “n” signifies the number of animals and “N” signifies the number of cells.

## 3 Results

### 3.1 Hippocampal volume is decreased in V-Lep^○b^-/- mice

To examine for structural differences in the hippocampus between middle-aged wild-type (wt) and transgenic V-Lep^○b^-/- mice (tg), we conducted magnetic resonance imaging (MRI) in 6–12 month-old mice (Figure 1). No difference was evident between the hemispheres within each genotype (Fisher's LSD: wt-rightHC vs. wt-leftHC: *p* = 0.16; tg-rightHC vs. tg-leftHC: *p* = 0.76). However, the volumes of the right and the left hippocampi of V-Lep^○b^-/- mice (*n* =13, *N* = 9) were significantly smaller compared to wt hippocampi [*n* =13, *N* = 9; Figure 1C; two-way ANOVA: Genotype: *F*_(1,48)_ = 17.19, *p* = 0.0001, hemisphere: *F*_(1,48)_ = 0.1.54, *p* = 0.22; *post-hoc* Fisher's LSD: wt-rightHC vs. tg-rightHC: *p* = 0.022; wt-leftHC vs. tg-leftHC: *p* = 0.001].

### 3.2 LTP is impaired in V-Lep^○b^-/- mice

**Figure 2:**
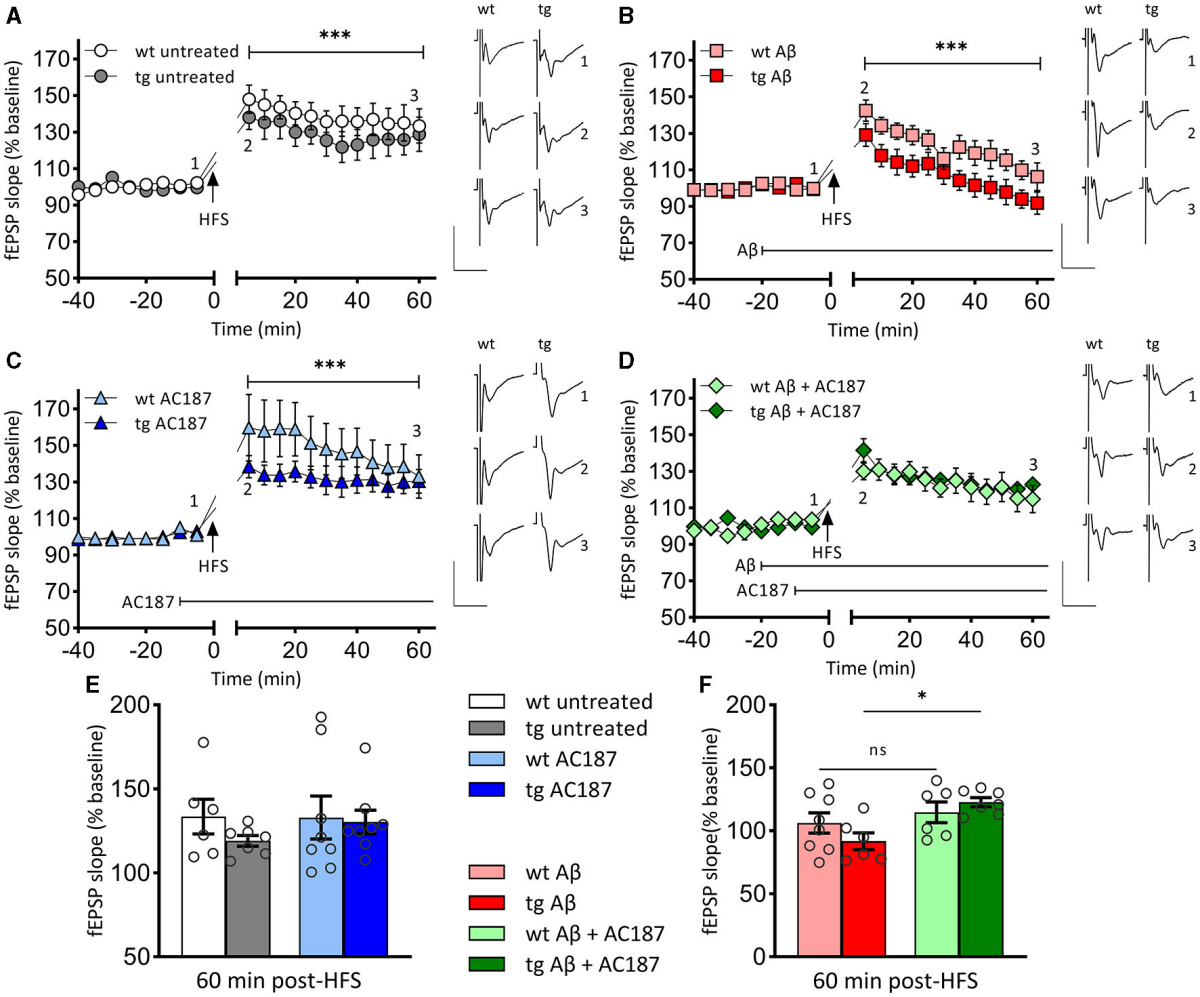
LTP that is impaired by Aβ (1–42)-treatment, is rescued in V-Lep^○b^-/- mice by amylin receptor antagonism. **(A)** Robust LTP that lasts for >60 min is induced by high-frequency stimulation (HFS) of both V-Lep^○b^-/- mice (tg; *n* = 5, *N* = 8) and their wild-type littermates (wt; *n* = 5, *N* = 6). LTP in wildtype animals is slightly increased [ANOVA: *F*_(7,77)_ = 117.0, *p* < 0.0001; *post-hoc* Fisher's LSD, *p* < 0.0001]. **(B)** Compared to control responses shown in **(A)**, treatment with oligomeric Aβ (1–42) significantly impairs LTP in both tg (*n* = 5, *N* = 8) and wt (*n* = 5, *N* = 6) mice [ANOVA: *F*_(7,77)_ = 117.0, *p* < 0.0001; *post-hoc* Fisher's LSD, *p* < 0.0001]. Effects are also significantly more potent in tg compared to wt hippocampi (*post-hoc* Fisher's LSD, *p* < 0.0001). **(C)** LTP is significantly enhanced by treatment with an amylin receptor antagonist (AC187) in both tg [*n* = 5, *N* = 8; ANOVA: *F*_(7,77)_ = 117.0, *p* < 0.0001; *post-hoc* Fisher's LSD, *p* = 0.042] and wt (*n* = 8, *N* = 8; *post-hoc* Fisher's LSD, *p* < 0.0001), mice, compared to untreated animals. **(D)** Subsequent treatment with AC187 rescues LTP deficits caused by Aβ(1–42) in tg [*n* = 5, *N* = 7; ANOVA: *F*_(7,77)_ = 117.0, *p* < 0.0001; *post-hoc* Fisher's LSD, *p* < 0.0001] but not wt mice (*n* = 5, *N* = 6; *post-hoc* Fisher's LSD, *p* = 0.58). **(E)** Net effect of treatments displayed as average fEPSP at 60 min post HFS (three-factor two-way ANOVA [Interaction: *F*_(3,48)_ = 0.8, *p* = 0.5; Genotype: *F*_(1,48)_ = 0.98, *p* = 0.33, Treatment: *F*_(3,48)_ = 6.03, *p* = 0.0014]. No statistical difference is evident between the genotypes for either the untreated (*post-hoc* Fisher's LSD, *p* = 0.244), or AC187 treatment conditions (*post-hoc* Fisher's LSD, *p* = 0.815). One outlier not shown in wt untreated. **(F)** Oligomeric Aβ(1–42) application reduces the average fEPSP for both genotypes (wt: *post-hoc* Fisher's LSD, *p* = 0.026; tg: *post-hoc* Fisher's LSD, *p* = 0.03). In transgenic animals the debilitating effect of Aβ(1–42) was rescued by subsequent application of AC187 (*post-hoc* Fisher's LSD, *p* = 0.0145). Analogs show representative fEPSPs signified by the respective numbering in the graphs. Vertical scale bars: 1 mV, horizontal scale bars: 10 ms. Data are displayed as mean ± SEM. Significance levels, ns *p* >0.05, *p < 0.05, ***p < 0.001.

Having found a difference in hippocampal volumes, we then explored whether these deficits would be reflected by differences in hippocampal LTP in wt and tg mice (Figure 2). High frequency stimulation (HFS) of Schaffer collaterals resulted in LTP of CA1 stratum radiatum synapses in wt (*n* = 5, *N* = 6) and tg mice (*n* = 5, *N* = 8) that persisted for >1 h post HFS in both groups (Figure 2A).

LTP was significantly different between wt and tg animals [ANOVA: *F*_(7,77)_ = 117.0, *p* < 0.0001], whereby LTP in tg animals was impaired compared to wt littermates (Figure 2A; *post-hoc* Fisher's LSD, *p* < 0.0001]. Membrane excitability was equivalent in both groups: comparison of stimulus-response properties in wt and tg hippocampi revealed no differences [unpaired *t*-test (*t* = 0.3092, df = 22) *p* = 0.76; stimulus intensity 50–600 μA, in steps of 50 μA, wt: *n* = 12, *N* = 16; tg: *n* = 10, *N* = 23, data not shown].

### 3.3 Aβ(1–42) causes impairments in LTP that are more severe in V-Lep^○b^-/- mice

We next explored whether differences in the sensitivity of the hippocampus to oligomeric Aβ(1–42) might occur in V-Lep^○b^-/- mice. HFS in the presence of Aβ(1–42) resulted in an impairment of LTP in wt animals (*n* = 5, *N* = 8) compared to LTP in untreated wt [Figures 2A, B; ANOVA: *F*_(7,77)_ = 117.0, *p* < 0.0001; *post-hoc* Fisher's LSD, *p* < 0.0001]. LTP was also impaired in Aβ(1–42)-treated tg mice (*n* = 5, *N* = 6) compared to LTP in untreated tg hippocampus (Figures 2A, B; tg untreated vs. tg Aβ: *post-hoc* Fisher's LSD, *p* < 0.0001]. In the latter case, evoked potentials had returned to baseline levels by 60 min after HFS. We also detected a significantly poorer LTP profile in Aβ(1–42)-treated tg animals compared to Aβ(1–42)-treated wt animals that was apparent from 5 min post-HFS onwards (Figure 2B; *post-hoc* Fisher's LSD, *p* < 0.0001). This indicates that V-Lep^○b^-/- tg mice are more vulnerable to the effects of Aβ(1–42) on hippocampal LTP than their wt littermates.

### 3.4 Amylin receptor antagonism enhances LTP in wildtype and V-Lep^○b^-/- mice

Next, we explored to what extent application of an amylin receptor antagonist can alter the profile of hippocampal LTP in V-Lep^○b^-/- tg mice and their wt littermates.

AC187 (250 nm) significantly increased LTP in wt mice (*n* = 8, *N* = 8) compared to LTP induced in the absence of treatment [Figures 2A, C; ANOVA: *F*_(7,77)_ = 117.0, *p* < 0.0001; *post-hoc* Fisher's LSD, *p* < 0.0001], whereby effects on early LTP were most pronounced. When applied to hippocampal slices from V-Lep^○b^-/- tg mice (*n* = 5, *N* = 8), AC187 also elicited an increase in LTP compared to untreated LTP responses in the tg animals (Figures 2A, C; *post-hoc* Fisher's LSD, *p* = 0.042). Thus, amylin receptor antagonism improves LTP in V-Lep^○b^-/- tg mice and their wt littermates.

### 3.5 Amylin receptor antagonism prevents LTP impairments by aβ(1–42) in V-Lep^○b^-/- mice

Having, found impairments of LTP by Aβ(1–42) in both tg and wt mice, we assessed to what extent treatment of hippocampal slices with an amylin antagonist could restore LTP (Figure 2D).

Here, we detected that treatment with AC187 (250 nm) elicited a recovery of LTP in Aβ(1–42) -treated tg hippocampi when compared to LTP induced in tg slices (*n* = 5, *N* = 7), in the presence of Aβ(1–42) alone [ANOVA: *F*_(7,77)_ = 117.0, *p* < 0.0001; *post-hoc* Fisher's LSD, *p* < 0.0001]. By contrast, AC187 treatment had no effect on LTP in Aβ(1–42)-treated wt hippocampi (*n* = 5, *N* = 6), compared to wt slices treated with Aβ(1–42) alone (*post-hoc* Fisher's LSD, *p* = 0.58).

Thus, amylin receptor antagonism rescues Aβ(1–42)-mediated impairments of LTP in V-Lep^○b^-/- tg but not wt mice.

The beneficial effects of amylin antagonism on LTP in Aβ(1–42)-treated tg hippocampi appeared to particularly target the later phase of LTP. To assess this, we compared fEPSP responses 60 min post-HFS in wt and tg hippocampi [Figures 2E, F; three-factor two-way ANOVA (Interaction: *F*_(3,48)_ = 0.8, *p* = 0.5; Genotype: *F*_(1,48)_ = 0.98, *p* = 0.33, Treatment: *F*_(3,48)_ = 6.03, *p* = 0.0014]. The magnitude of potentiation was equivalent in untreated wt and tg slices (Figure 2E, *p* = 0.244), in AC187-treated (Figure 2E, *p* = 0.815), and in Aβ(1–42)-treated (Figure 2F, *p* = 0.228), tg and wt hippocampi, as well as in tg and wt hippocampi that were treated with both AC187 and Aβ(1–42; Figure 2F, *p* = 0.516; *post-hoc* Fisher's LSD). By contrast, LTP was significantly improved in tg slices that were treated with both AC187 and Aβ(1–42) compared to tg slices that were treated with only Aβ(1–42; *post-hoc* Fisher's LSD, *p* = 0.0145).

### 3.6 V-Lep^○b^-/- mice show altered passive and active membrane properties in hippocampal neurons

The formation and stability of LTP depends on the resting membrane potential, the threshold, and the firing frequency of neurons (Debanne and Russier, 2019). Changes in these passive and active cell properties can contribute to deficits in the induction and persistency of LTP (Südkamp et al., 2012). To examine if Aβ(1–42) affects cell properties of CA1 hippocampal neurons, we carried out whole cell patch clamp recordings in V-Lep^○b^-/- tg and wt mice under the same treatment conditions used in the LTP study (Table 3).

**Table 3:** Outcome of statistical analysis of active and passive electrophysiological properties of CA1 pyramidal neurons.

**Tested parameter**	**Applied test**	**Factors**	***F*_(DFn, DFd)_**	***P*-value**	***Post-hoc* multiple comparison**	**Selected comparisons and corresponding *p*-values**
Input resistance (MΩ)	Multifactor two-way ANOVA	Interaction	*F*_(3,167)_ = 0.5337	*P* = 0.6598	Uncorrected Fisher's LSD test	wt:untreated vs. wt:Aβ: **0.0445**
		Genotype	*F*_(1,167)_ = 5.246	*P* = 0.0232		wt:untreated vs. wt:AC187: **0.0015**
		Treatment	*F*_(3,167)_ = 15.53	*P* < 0.0001		wt:untreated vs. wt:Aβ + AC187: 0.5326
						wt:untreated vs. tg:untreated: 0.5766
						tg:untreated vs. tg:Aβ: **0.0003**
						tg:untreated vs. tg:AC187: **0.0004**
						tg:untreated vs. tg:Aβ + AC187: 0.7846
						wt:Aβ vs. wt:Aβ + AC187: **0.0089**
						wt:Aβ vs. wt: AC187: 0.2935
						tg:Aβ vs. tg:Aβ + AC187: **0.0001**
						tg:Aβ vs. tg: AC187: 0.7402
Membrane time constant (ms)	Multifactor two-way ANOVA	Interaction	*F*_(3,169)_ = 8.083	*P* < 0.0001	Uncorrected Fisher's LSD test	wt:untreated vs. wt:Aβ: 0.2538
		Genotype	*F*_(1,169)_ = 1.111	*P* = 0.2934		wt:untreated vs. wt:AC187: 0.6437
		Treatment	*F*_(3,169)_ = 38.04	*P* < 0.0001		wt:untreated vs. wt:Aβ + AC187: **<0.0001**
						wt:untreated vs. tg:untreated: **<0.0001**
						tg:untreated vs. tg:Aβ: **<0.0001**
						tg:untreated vs. tg:AC187: **<0.0001**
						tg:untreated vs. tg:Aβ + AC187: 0.1831
						wt:Aβ vs. wt:Aβ + AC187: **<0.0001**
						wt:Aβ vs. wt: AC187: 0.1044
						tg:Aβ vs. tg:Aβ + AC187: **<0.0001**
						tg:Aβ vs. tg: AC187: **<0.0001**
Resting potential (mV)	Multifactor two-way ANOVA	Interaction	*F*_(3,171)_ = 3.852	*P* = 0.0106	Uncorrected Fisher's LSD test	wt:untreated vs. wt:Aβ: **0.0008**
		Genotype	*F*_(1,171)_ = 6.353	*P* = 0.0126		wt:untreated vs. wt:AC187: 0.5137
		Treatment	*F*_(3,171)_ = 19.81	*P* < 0.0001		wt:untreated vs. wt:Aβ + AC187: 0.5759
						wt:untreated vs. tg:untreated: **0.0080**
						tg:untreated vs. tg:Aβ: **<0.0001**
						tg:untreated vs. tg:AC187: **<0.0001**
						tg:untreated vs. tg:Aβ + AC187: 0.6703
						wt:Aβ vs. wt:Aβ + AC187: **<0.0001**
						wt:Aβ vs. wt: AC187: **0.004**
						tg:Aβ vs. tg:Aβ + AC187: **<0.0001**
						tg:Aβ vs. tg: AC187: 0.6525
Threshold (pA)	Multifactor two-way ANOVA	Interaction	*F*_(3,168)_ = 6.582	*P* = 0.0003	Uncorrected Fisher's LSD test	wt:untreated vs. wt:Aβ: 0.5043
		Genotype	*F*_(1,168)_ = 21.40	*P* < 0.0001		wt:untreated vs. wt:AC187: 0.1668
		Treatment	*F*_(3,168)_ = 5.835	*P* = 0.0008		wt:untreated vs. wt:Aβ + AC187: **0.0469**
						wt:untreated vs. tg:untreated: 0.4219
						tg:untreated vs. tg:Aβ: **<0.0001**
						tg:untreated vs. tg:AC187: 0.5471
						tg:untreated vs. tg:Aβ + AC187: **0.0004**
						wt:Aβ vs. wt:Aβ + AC187: **0.0103**
						wt:Aβ vs. wt: AC187: **0.0442**
						tg:Aβ vs. tg:Aβ + AC187: 0.4177
						tg:Aβ vs. tg: AC187: **0.0002**
Spike amplitude (mV)	Multifactor two-way ANOVA	Interaction	*F*_(3,170)_ = 0.8002	*P* = 0.4954	Uncorrected Fisher's LSD test	wt:untreated vs. wt:Aβ: 0.8473
		Genotype	*F*_(1,170)_ = 12.12	*P* = 0.0006		wt:untreated vs. wt:AC187: 0.4486
		Treatment	*F*_(3,170)_ = 1.474	*P* = 0.2235		wt:untreated vs. wt:Aβ + AC187: 0.7215
						wt:untreated vs. tg:untreated: 0.2830
						tg:untreated vs. tg:Aβ: 0.3318
						tg:untreated vs. tg:AC187: 0.3590
						tg:untreated vs. tg:Aβ + AC187: 0.2078
						wt:Aβ vs. wt:Aβ + AC187: 0.8860
						wt:Aβ vs. wt: AC187: 0.6054
						tg:Aβ vs. tg:Aβ + AC187: 0.7945
						tg:Aβ vs. tg: AC187: 0.0593
Time to peak (ms)	Multifactor two-way ANOVA	Interaction	*F*_(3,169)_ = 2.992	*P* = 0.0325	Uncorrected Fisher's LSD test	wt:untreated vs. wt:Aβ: 0.0845
		Genotype	*F*_(1,169)_ = 7.082	*P* = 0.0085		wt:untreated vs. wt:AC187: 0.5458
		Treatment	*F*_(3,169)_ = 16.18	*P* < 0.0001		wt:untreated vs. wt:Aβ + AC187: **0.0073**
						wt:untreated vs. tg:untreated: **0.0342**
						tg:untreated vs. tg:Aβ: 0.2749
						tg:untreated vs. tg:AC187: **0.0002**
						tg:untreated vs. tg:Aβ + AC187: 0.0661
						wt:Aβ vs. wt:Aβ + AC187: **<0.0001**
						wt:Aβ vs. wt: AC187: 0.2216
						tg:Aβ vs. tg:Aβ + AC187: **0.0038**
						tg:Aβ vs. tg: AC187: **0.0091**
Peak to AHP (ms)	Multifactor two-way ANOVA	Interaction	*F*_(3,165)_ = 1.992	*P* = 0.1172	Uncorrected Fisher's LSD test	wt:untreated vs. wt:Aβ: 0.8016
		Genotype	*F*_(1,165)_ = 5.022	*P* = 0.0264		wt:untreated vs. wt:AC187: 0.8109
		Treatment	*F*_(3,165)_ = 16.95	*P* < 0.0001		wt:untreated vs. wt:Aβ + AC187: **0.0097**
						wt:untreated vs. tg:untreated: 0.1545
						tg:untreated vs. tg:Aβ: 0.1218
						tg:untreated vs. tg:AC187: 0.0902
						tg:untreated vs. tg:Aβ + AC187: **0.0001**
						wt:Aβ vs. wt:Aβ + AC187: **0.0069**
						wt:Aβ vs. wt: AC187: 0.9725
						tg:Aβ vs. tg:Aβ + AC187: **<0.0001**
						tg:Aβ vs. tg: AC187: 0.9342
AHP depth (mV)	Multifactor two-way ANOVA	Interaction	*F*_(3,169)_ = 2.088	*P* = 0.1036	Uncorrected Fisher's LSD test	wt:untreated vs. wt:Aβ: 0.4791
		Genotype	*F*_(1,169)_ = 0.2837	*P* = 0.5950		wt:untreated vs. wt:AC187: 0.2155
		Treatment	*F*_(3,169)_ = 0.9503	*P* = 0.4177		wt:untreated vs. wt:Aβ + AC187: 0.0888
						wt:untreated vs. tg:untreated: **0.0480**
						tg:untreated vs. tg:Aβ 0.0697
						tg:untreated vs. tg:AC187: 0.9596
						tg:untreated vs. tg:Aβ + AC187: 0.1360
						wt:Aβ vs. wt:Aβ + AC187: 0.3665
						wt:Aβ vs. wt: AC187: 0.6610
						tg:Aβ vs. tg:Aβ + AC187: 0.7144
						tg:Aβ vs. tg: AC187: 0.0527
AP half width (ms)	Multifactor two-way ANOVA	Interaction	*F*_(3,169)_ = 4.098	*P* = 0.0077	Uncorrected Fisher's LSD test	wt:untreated vs. wt:Aβ: **0.0419**
		Genotype	*F*_(1,169)_ = 6.800	*P* = 0.0099		wt:untreated vs. wt:AC187: **0.0191**
		Treatment	*F*_(3,169)_ = 34.81	*P* < 0.0001		wt:untreated vs. wt:Aβ + AC187: **0.0059**
						wt:untreated vs. tg:untreated: **<0.0001**
						tg:untreated vs. tg:Aβ: **<0.0001**
						tg:untreated vs. tg:AC187: **<0.0001**
						tg:untreated vs. tg:Aβ + AC187: 0.8191
						wt:Aβ vs. wt:Aβ + AC187: **<0.0001**
						wt:Aβ vs. wt: AC187: 0.9122
						tg:Aβ vs. tg:Aβ + AC187: **<0.0001**
						tg:Aβ vs. tg: AC187: 0.6622
Total spike time (ms)	Multifactor two-way ANOVA	Interaction	*F*_(3,155)_ = 6.632	*P* = 0.0003	Uncorrected Fisher's LSD test	wt:untreated vs. wt:Aβ: 0.2376
		Genotype	*F*_(1,155)_ = 23.71	*P* < 0.0001		wt:untreated vs. wt:AC187: 0.3358
		Treatment	*F*_(3,155)_ = 24.24	*P* < 0.0001		wt:untreated vs. wt:Aβ + AC187: 0.0631
						wt:untreated vs. tg:untreated: **<0.0001**
						tg:untreated vs. tg:Aβ: **<0.0001**
						tg:untreated vs. tg:AC187: **<0.0001**
						tg:untreated vs. tg:Aβ + AC187: 0.1274
						wt:Aβ vs. wt:Aβ + AC187: **<0.0001**
						wt:Aβ vs. wt: AC187: 0.7518
						tg:Aβ vs. tg:Aβ + AC187: **<0.0001**
						tg:Aβ vs. tg: AC187: 0.4926

The table shows the outcome of a multifactorial two-way ANOVA to assess the conditions of genotype, treatment and interaction in tg and wt animals that were untreated, or treated with Aβ(1–42), AC187, or AC187 in the presence of Aβ(1–42). The parameters tested are listed in the first column from the left. In addition, the outcome of *post-hoc* multiple comparisons using Fisher's LSD-test are described (first and second columns from the right). Significant differences are highlighted in bold.

**Figure 3:**
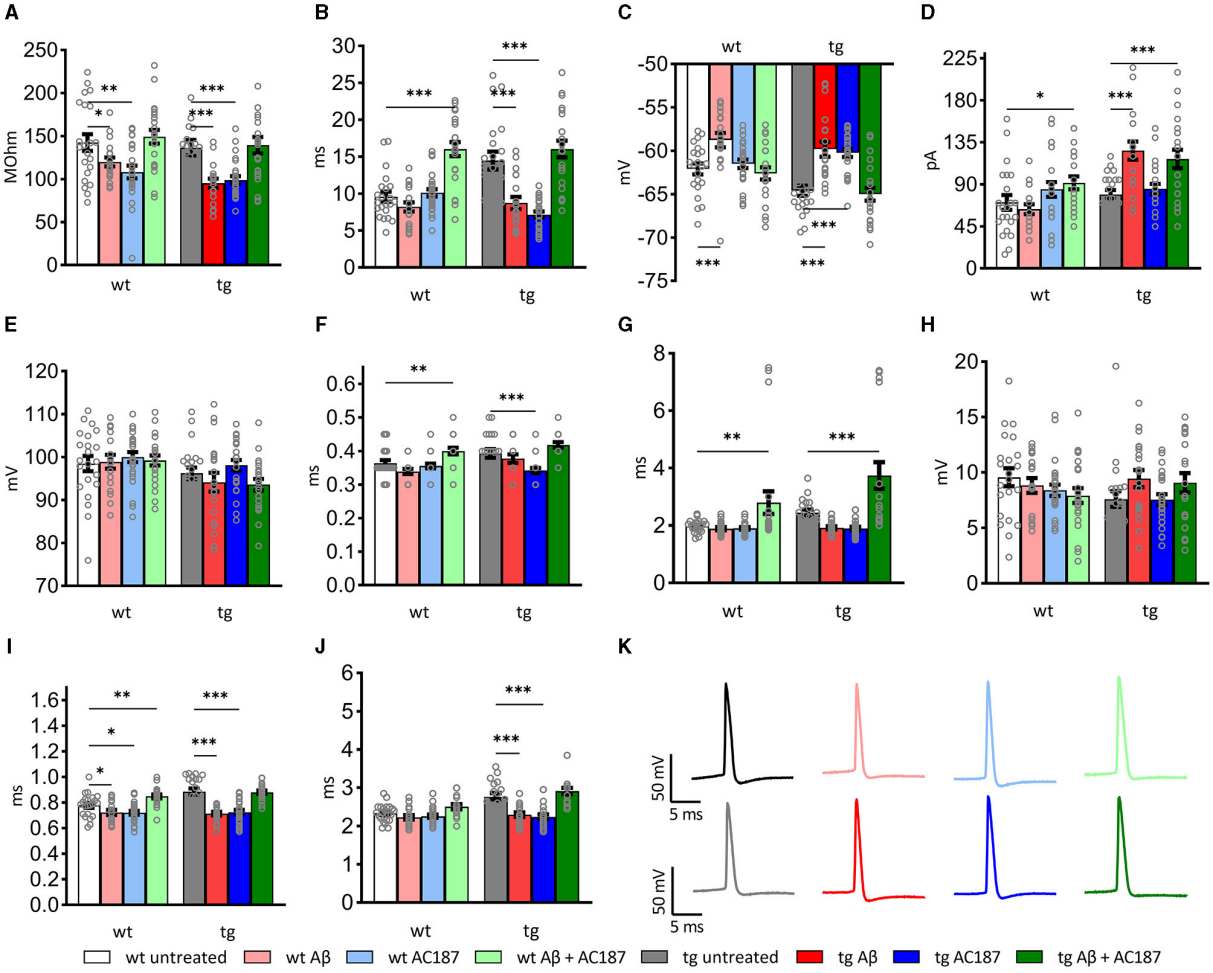
Passive and active electrophysiological properties of hippocampal CA1 cells of wildtype (wt; *n* = 8, *N* = 22), and V-Lep^○b^-/- (tg) animals (*n* = 12, *N* = 22) in the presence of Aβ (1–42; wt: *n* = 5, *N* = 19; tg: *n* = 5, *N* = 20) or amylin receptor antagonism (wt: *n* = 5, *N* = 25; tg: *n* =5, *N* = 26) and in an additional application of both substances (wt: *n* = 6, *N* = 23; tg: *n* = 5, *N* = 22). Statistical analysis was done by applying a multi-factoral two-way ANOVA followed by a Fisher's LSD test for multiple comparisons. Individual statistics details are described in Table 3. **(A)** Oligomeric Aβ (1–42) application reduces the input resistance; amylin receptor antagonism rescues this effect in wt and tg animals (wt untreated vs. wt Aβ: *p* = 0.05; tg untreated vs. tg Aβ: *p* = 0.0003). **(B)** The tau of transgenic neurons is significantly enhanced compared to wt (wt untreated vs. tg untreated: *p* = 0.008, *p* < 0.0001, respectively). Treatment with oligomeric Aβ(1–42) alters tau in tg neurons (tg untreated vs. tg Aβ: *p* < 0.0001). Effects are rescued by AC187 application (tg Aβ vs. tg Aβ+AC187: *p* < 0.0001). In wt neurons Aβ (1–42) has no effect (wt untreated vs. wt Aβ: *p* = 0.25), although application of AC187 in the presence of Aβ (1–42) increases wt tau significantly (wt untreated vs. wt Aβ+AC187: *p* < 0.0001). **(C)** In both genotypes, membrane potentials were significantly increased by Aβ(1–42) treatment (wt *p* = 0.0008; tg *p* < 0.0001). Application of AC187 in the presence of Aβ(1–42) restores this effect to untreated conditions (tg untreated vs. tg Aβ+AC187: *p* = 0.67; wt untreated vs. wt Aβ + AC187: *p* = 0.5759). **(D)** Oligomeric Aβ (1–42)-treatment significantly increases threshold current in tg and has no effect in wt neurons (wt *p* = 0.5; tg *p* < 0.0001). Application of AC187 in the presence of Aβ (1–42) increases wt threshold current (wt Aβ vs. wt Aβ + AC187: *p* = 0.0103) and does not rescue increased threshold currents in tg neurons (tg Aβ vs. tg Aβ + AC187: *p* = 0.4177). **(E)** Spike amplitudes of both genotypes were unaffected by treatment with Aβ (1–42; wt *p* = 0.85; tg *p* = 0.36), or application of AC187 in the presence of Aβ(1–42; wt Aβ vs. wt Aβ + AC187: *p* = 0.6; tg Aβ vs. tg Aβ + AC187: *p* = 0.8). **(F)** The time to peak was heterogeneously affected in the two genotypes by Aβ (1–42) application, or amylin receptor antagonism. Aβ (1–42), or AC187 had no effect in wt neurons, but application of AC187 in the presence of Aβ(1–42) increased responses (wt untreated vs. wt Aβ + AC187: *p* = 0.0073). In tg animals, AC187 treatment reduced the time to peak (tg untreated vs. tg AC187: *p* = 0.0002). Application of Aβ (1–42) alone, or AC187 in the presence of Aβ (1–42) had no effect compared to untreated controls. **(G)** The time to after hyperpolarisation peak was only affected by application of AC187 in the presence of Aβ (1–42) in both genotypes (wt untreated vs. wt Aβ + AC187: *p* = 0.0097; tg untreated vs. tg Aβ + AC187: *p* = 0.0001). **(H)** The depth of the after hyperpolarisation (AHP) was unaffected by the different treatment conditions in tg and wt mice. **(I)** In wt animals, the action potential (AP) half-width was reduced by treatments with either Aβ (1–42) or AC187, and increased when AC187 was applied in the presence of Aβ (1–42; wt untreated vs. wt Aβ: *p* = 0.0419; wt untreated vs. wt AC187: *p* = 0.0191; wt untreated vs. wt Aβ + AC187: *p* = 0.0059). In tg neurons sole application of either Aβ(1–42), or AC187, reduced the AP half-width (tg untreated vs. tg Aβ: *p* < 0.0001; tg untreated vs. tg AC187: *p* < 0.0001). When AC137 was applied in the presence of Aβ (1–42), control levels were restored (tg untreated vs. tg Aβ + AC187: *p* = 0.8191). **(J)** In wt neurons the total spike time was unaffected by either of the treatments, whereas tg neurons showed reduced timings following application of either Aβ (1–42) or AC187 (tg untreated vs. tg Aβ: *p* < 0.0001; tg untreated vs. tg AC187: *p* < 0.0001). Application of AC187 in the presence of Aβ(1–42) restored the total spike time back to control levels in tg animals (tg untreated vs. tg Aβ + AC187: *p* = 0.1274). **(K)** Analog examples of action potentials recorded in wt (top row) and tg (bottom row), whereby gray analogs correspond to responses recorded the untreated condition, red shades correspond to Aβ (1–42)-treatment, blue shades to AC187 treatment and green shades to dual treatment with Aβ (1–42) and AC187. Data are shown as mean ± SEM. Significance levels, ns *p* > 0.05, *p < 0.05, **p < 0.01, ***p < 0.001.

Here, compared to wt control slices (*n* = 8, *N* = 22), both the resting membrane potential and the membrane time constant were significantly higher in control tg slices (*n* = 12, *N* = 22, *post-hoc* Fisher's LSD test: wt untreated vs. tg untreated: *p* = 0.008, *p* < 0.0001, respectively). The input resistance of both genotypes' neurons did not differ, however (*p* = 0.58; Figures 3A–C).

When we examined active membrane properties (Figures 3D–J), although we found no difference between the current threshold (*p* = 0.42) and the spike amplitude (*p* = 0.28) the timings of the action potential (AP) were significantly affected by the difference in genotype. Hippocampal neurons of V-Lep^○b^-/- tg mice exhibited a longer time to peak compared to wt neurons (*p* = 0.03), their AP half-width was longer (*p* < 0.0001) and their total spike time (*p* < 0.0001) was increased compared to wildtype cells. Although the time to AP peak (*p* = 0.034 and the after-hyperpolarisation (AHP) were similar (*p* = 0.16), the AHP this latter effect could lead to a depth was significantly shallower in tg compared to wt neurons (*p* = 0.048).

### 3.7 Both passive and active electrophysiological properties of hippocampal neurons are altered by aβ (1–42). Effects are more pronounced in V-Lep^○b^-/- mice

The application of Aβ(1–42) had no effect on spike amplitude (Figure 3E; Fisher's LSD: wt untreated vs. wt Aβ: *p* = 0.85; tg untreated vs. tg Aβ: *p* = 0.33), the time to peak (Figure 3F; wt untreated vs. wt Aβ: *p* = 0.09; tg untreated vs. tg Aβ: *p* = 0.28), the Peak to AHP (Figure 3G; wt *p* = 0.8; tg *p* = 0.12) and the amplitude of the AHP (depth; Figure 3H; wt *p* = 0.48; tg *p* = 0.07) in wildtype (*n* = 5, *N* = 19) and tg slices (*n* = 5, *N* = 20).

By contrast, significant effects of Aβ(1–42) were evident with regard to input resistance (Figure 3A; wt untreated vs. wt Aβ: *p* = 0.05; tg untreated vs. tg Aβ: *p* = 0.0003), membrane potential (Figure 3C; wt *p* = 0.0008; tg *p* < 0.0001) and AP half-width (Figure 3I; wt *p* = 0.04, tg *p* < 0.0001). The remaining three tested parameters were only affected by Aβ(1–42) in neurons from transgenic animals. Here, we observed that Aβ(1–42) treatment affected the membrane time constant, tau, in transgenic, but not in wildtype neurons (Figure 3B; uncorrected Fisher's LSD test: wt untreated vs. wt Aβ: *p* = 0.25; tg untreated vs. tg Aβ: *p* < 0.0001). The threshold current (Figure 3D) was reduced by Aβ(1–42) application to tg slices and required an increased current to reach the threshold, whereas wt-cells were unaffected (wt untreated vs. wt Aβ: *p* = 0.5; tg untreated vs. tg Aβ: *p* < 0.0001). Total spike time was unaffected by Aβ(1–42) treatment of wildtype neurons, whereas neurons of transgenic hippocampus showed significantly reduced total spike time after Aβ(1–42) application (Figure 3J; wt untreated vs. wt Aβ: *p* = 0.24; tg untreated vs. tg Aβ: *p* < 0.0001). In summary, those electrophysiological parameters that were affected by Aβ(1–42)-treatment in wt neurons, were also affected in tg neurons. However, V-Lep^○b^-/- tg hippocampi were more sensitive to Aβ(1–42) than wt hippocampi.

### 3.8 Amylin receptor antagonism decreases the membrane time constant, the membrane potential and AP timings in V-Lep^○b^-/- but not wildtype mice

We then tested to what extent amylin antagonism using AC187 affects V-Lep^○b^-/- tg (*n* =5, *N* = 26) and wt cells (*n* = 5, *N* = 25). Here we made the interesting observation that several active neuronal properties of tg mice were significantly affected by amylin receptor antagonism, although wt slices were unaffected.

For example, tg, but not wt, neurons exhibited a significantly decreased membrane time constant (tau; Figure 3B; Fisher's LSD: wt untreated vs. wt AC187: *p* = 0.64; tg untreated vs. tg AC187: *p* < 0.0001), and a significantly less negative membrane potential (Figure 3C; wt untreated vs. wt AC187: *p* = 0.51; tg untreated vs. tg AC187: *p* < 0.0001) compared to untreated responses. Furthermore, the time to peak (Figure 3F; wt: *p* = 0.55; tg: *p* = 0.0002) as well as the total spike time (Figure 3J; wt: *p* = 0.34; tg: *p* < 0.0001) were significantly decreased in tg, but not wt hippocampi following antagonist treatment.

Amylin antagonism had no effect on the current threshold (Figure 3D; wt: *p* = 0.17; tg: *p* = 0.55) or the spike amplitude (Figures 3E, K; wt: *p* = 0.45; tg: *p* = 0.36) in either genotype. By contrast, both the input resistance (Figure 3A) and the AP half-width (Figure 3I) were reduced in both genotypes following the application of AC187 (R_in_ wt: *p* = 0.0015; tg: *p* = 0.0004; AP half-width: wt: *p* = 0.02; tg: *p* < 0.0001).

### 3.9 Amylin receptor antagonism alters passive and active cell responses in aβ (1–42)-treated V-Lep^○b^-/- and wildtype mice

Next, we tested cell responses in the hippocampus when AC187 was applied in the presence of Aβ(1–42). Here, input resistance was improved in both tg (*n* = 5, *N* = 22) and wt (*n* = 6, *N* = 23) mice (uncorrected Fisher's LSD test: wt untreated vs. wt Aβ+AC187: *p* = 0.53; tg untreated vs. tg Aβ+AC187: *p* = 0.79; Figure 3A). Treatment with AC187 specifically restored deficits that had been caused by Aβ(1–42)-treatment of tg slices: it counteracted the reductions of tau elicited by Aβ(1–42; tg Aβ vs. tg Aβ+AC187: *p* < 0.0001; Figure 3B), the membrane potential of tg cells was also improved back to untreated levels (Figure 3C; tg untreated vs. tg Aβ+AC187: *p* = 0.67). Furthermore, the AP half-width of tg cells was also restored in tg neurons back to untreated levels (Figure 3I; tg untreated vs. tg Aβ+AC187: *p* = 0.82) as was the total spike time (Figure 3J; tg untreated vs. tg Aβ+AC187: *p* = 0.13).

A few active neuronal properties were negatively affected in the genotypes by the combined application of AC187 with Aβ(1–42). In those cases, the outcome exceeded the individual effects of either Aβ(1–42) or AC187 treatment. For example, although the membrane time constant (tau) of wt animals, was unaffected by Aβ(1–42) or AC187, joint application of the peptides significantly increased tau, to well above untreated levels (uncorrected Fisher's LSD test: wt untreated vs. wt Aβ+AC187: *p* < 0.0001; Figure 3B). A similar effect was found in wt neurons with regard to time to peak (wt untreated vs. wt Aβ+AC187: *p* = 0.007; Figure 3F). Another parameter that was more potential affected by the combined presence of AC187 and Aβ(1–42) was the peak to after-hyperpolarisation duration. Here although sole application of Aβ(1–42) or AC187 had no effect in either wt or tg slices, the combined peptide application was resulted in a significantly increased peak to AHP duration compared to untreated levels (Figure 3G; wt untreated vs. wt Aβ+AC187: *p* = 0.01; tg untreated vs. tg Aβ+AC187: *p* = 0.0001; Results and statistical analyses of all conditions are summarized in Table 3).

## 4 Discussion

In this study we examined hippocampal changes in a mouse model of T2D. We observed that in the V-Lep^○b^-/- transgenic mouse, hippocampal volume is significantly reduced, and that LTP in the hippocampus is impaired compared to wild-type conditions. Furthermore, the hippocampus of the transgenic mouse is more vulnerable to debilitation by Aβ(1–42), and amylin receptor antagonism restores LTP to control levels. Patch clamp recordings revealed a potent reduction in firing frequency of hippocampal neurons of V-Lep^○b^-/- mice in response to Aβ(1–42), whereas firing frequency in wildtype mice was increased. Strikingly, amylin antagonism enhanced LTP in both strains, but amylin antagonism was more effective at restoring Aβ(1–42)-mediated debilitations of passive and active cell membrane properties in V-Lep^○b^-/- tg mice compared to wt littermates. Taken together, our data indicate that a T2D-like state increases the vulnerability of the hippocampus to debilitation by Aβ(1–42) and that these effects are mediated, not only by leptin deficiency, but also by altered amylin signaling.

The V-Lep^○b^-/- transgenic mouse presents many features of T2D such as hyperinsulinaemia, insulin resistance, leptin deficiency, hyperglycaemia and adipositas (Zechner, 2015). Hippocampal atrophy has been reported in the brains of middle-aged and elderly T2D patients (Schmidt et al., 2004; Milne et al., 2017; Schneider et al., 2017). Consistent with this, we detected significant reductions in hippocampal volume in 6–12 month-old V-Lep^○b^-/- mice. In contrast to studies in T2D patents that were in young middle age, where asymmetric hippocampal atrophy was reported (Milne et al., 2017), we detected equivalent losses of volume in both hippocampi. This may relate to the age of the mice at the time point of investigation given that 6–12 months of age in rodents corresponds to advanced middle age (Twarkowski and Manahan-Vaughan, 2016). Despite the loss of hippocampal volume, marked cognitive deficits were not apparent in our transgenic mice. Hippocampal synaptic plasticity was also unaffected. Thus, despite the loss of hippocampal volume, animals had no apparent hippocampus-dependent functional deficits. Despite this, we detected an increased sensitivity of the V-Lep^○b^-/- mice to LTP deficits mediated by oligomeric Aβ(1–42). Both wild-type littermates and transgenic animals exhibited impairments in the later phases of LTP, but effects were more prominent in the T2D mice.

This sensitivity may reflect changes in insulin receptor function in the brain in T2D. The hippocampus expresses insulin receptors in high density (Werther et al., 1987; Marks et al., 1990) and insulin acts via the insulin-IRS-Akt and MAPK pathway (van der Heide et al., 2006; Nelson et al., 2008). Whereas, the Akt pathway contributes to the regulation of bidirectional synaptic strength (van der Heide et al., 2005), the MAPK pathway is important for LTP (Miyamoto, 2006). Thus, changes in insulin sensitivity in the brain may not only lead to hypometabolism but may directly impact on signaling cascades that contribute to LTP. Acute treatment of the rodent brain with oligomeric Aβ(1–42) results in a potent impairment of LTP and changes in hippocampal neuronal oscillations (Walsh et al., 2002; Kalweit et al., 2015). AD pathology includes glucose hypometabolism (Walsh et al., 2002). This is evident even in cognitively normal ApoE4 individuals but is more pronounced in AD patients (Ryu et al., 2019). Changes in glucose uptake in presymptomatic AD indicates that this may contribute to disease pathology (Ryu et al., 2019). Our data suggests that this change may not be causative but will increase the vulnerability of the brain to Aβ (1–42)-mediated pathology.

Amylin is a 37-amino acid neuroendocrine polypeptide hormone that is co-secreted with insulin from pancreatic β-cells (Westermark et al., 2011; Bower and Hay, 2016). Aside from supporting the regulation of blood glucose levels, amylin contributes to neuronal development and energy metabolism and neuronal development (Hay et al., 2015; Levin and Lutz, 2017). Amylin crosses the blood brain barrier (Jarosz-Griffiths et al., 2016; Mietlicki-Baase, 2016) and amylin receptors are present in the brain (Beaumont et al., 1993; Sexton et al., 1994; Dunn-Meynell et al., 2016). Under physiological conditions, activation of amylin receptors results in downstream cellular signaling via the ERK1/2 and Akt pathways (Visa et al., 2015; Fu et al., 2017) but, under conditions where blood glucose levels are high, such as in T2D, these signaling pathways are inhibited by activation of amylin receptors (Visa et al., 2015). This may explain why wild-type littermates of V-Lep^○b^-/- transgenic mice responded with *improved* LTP to amylin antagonism: the Erk1/2 signaling cascade is involved in hippocampal long-term depression (LTD; Thiels et al., 2022). Thus, a reduction of the activity of this signaling cascade by amylin antagonism may act permissively toward improved LTP. By contrast in V-Lep^○b^-/- transgenic mice intrinsic increases in the release of insulin from pancreatic β-cells occur (Baribault, 2010) and amylin is co-secreted with insulin. Under these conditions insulin-supported induction of hippocampal LTP via activation of the PI3K/Akt pathway (Zhao et al., 2019) and the activation by amylin of the Erk and Akt pathways (Visa et al., 2015) may become dysfunctional. In this case amylin receptor antagonism may have served to normalize the contribution of the Erk and Akt pathways to LTP.

Oligomeric Aβ(1–42) inhibits N-methyl-D-aspartate receptor (NMDAR)-dependent hippocampal LTP in rodents (Selkoe, 2008; Li et al., 2011; Kalweit et al., 2015). In recent years it has become apparent that antagonism of amylin receptors in the brains of rodents with increased amyloid burden can improve debilitated hippocampal LTP (Kimura et al., 2012, 2017; Soudy et al., 2019). Inhibition of LTP by Aβ(1–42), or amylin is also prevented by amylin receptor antagonism (Soudy et al., 2016). It is thus, perhaps, not surprising that amylin receptor antagonism rescued LTP from the negative effects of oligomeric Aβ(1–42) in both V-Lep^○b^-/- transgenic mice and their wild-type littermates. What is curious is the acute efficacy of this treatment. Others have reported that intraperitoneal treatment of 6 months old 5XFAD mice with amylin receptor antagonists for 5 weeks improves hippocampal LTP and spatial learning, as well as reduced amyloid plaque burden and neuroinflammation (Soudy et al., 2019). The authors of the study propose that these effects may be mediated by neuroprotection, blockade of microglial amylin receptors (thereby reducing neuroinflammation (Fu et al., 2017), or Aβ efflux from the brain through increased expression of LRP1 (Zhu et al., 2015; Soudy et al., 2019). All of these effects are chronic, however. One possible explanation for the acute effects of amylin antagonism that we observed in the present study is that amylin interacts directly with Aβ(1–42): others have reported a high-affinity interaction of Aβ with amylin (Yan et al., 2014) and that amylin directly mediates the impairing effect of Aβ(1–42) on LTP (Kimura et al., 2012). Aβ(1–40) can bind to the cell membrane to form cation-selective pores (Sciacca et al., 2012). Ca^2+^ influx through these pores occurs as early as 10 min after application of Aβ (Sciacca et al., 2012). Specific changes in intracellular calcium are intrinsic to the induction of LTP and LTD (Cummings et al., 1996). Low elevations of intracellular Ca^2+^ concentrations activate phosphatases that support the induction of LTD (Mulkey et al., 1994; Cummings et al., 1996) and undermine LTP (Huang et al., 2001). Thus, the acute effects of amylin antagonism on Aβ-debilitated LTP may have resulted from an acute interference of Aβ-interactions with the cell membrane and the prevention of subsequent changes in Ca^2+^-dependent LTP mechanisms. This hypothesis is supported by the observation that it was the later phases of LTP that were affected by Aβ(1–40) and were rescued by amylin antagonism: Ca^2+^-mediated activation of phosphatases relevant for LTD, or kinases (e.g., CAMKII) relevant for LTP are important for plasticity processes downstream of the activation of the NMDAR (Soderling and Derkach, 2000; Lisman et al., 2012; Hell, 2016), that are required for the induction and very early phase of LTP (Volianskis et al., 2015).

Our scrutiny of changes in passive and active cellular properties in the transgenic and wildtype mice revealed that the resting membrane potential and the membrane time constant were higher, the time to peak of the action potential (AP) was longer and the total spike time was increased in tg compared to wt neurons. These differences did not play out in an altered LTP response in untreated tg hippocampi but may have contributed to the increased vulnerability of the transgenic hippocampus to Aβ(1–42). Here, we found that input resistance was lower, the membrane potential was less negative, and the AP half-width was reduced in both genotypes in the presence of Aβ(1–42). These properties could be expected to lower the threshold for hippocampal LTP induction and thus act permissively toward an improved LTP (Linden, 1999). This was not the case, however, and one possible explanation is that the action potential threshold was significantly higher in both Aβ(1–42)-treated transgenic compared to wildtype neurons, suggesting that the threshold for induction of LTP was also higher in Aβ(1–42)-treated V-Lep^○b^-/- mice. Amylin antagonism also affected cell properties of tg neurons. The membrane time constant, tau, was reduced, the AP time-to-peak and total spike time were decreased, and the membrane potential was less negative compared to untreated tg neurons. These properties were unaffected in wildtype neurons. The input resistance and the AP half-width were reduced in both genotypes (as was the case in the presence of Aβ(1–42). Given that LTP was improved in the presence of AC187 and impaired by Aβ(1–42), one possible explanation is that the improved AP properties in AC187-treated transgenic neurons formed the basis for this effect. When the amylin antagonist was applied in the presence of Aβ(1–42), it rescued most of the changes in neuronal properties that had been caused by Aβ(1–42)-treatment. One interesting facet was the potent increase in the time to peak of the after hyperpolarization that was evident in both genotype compared to all other test conditions, suggesting that amylin receptor antagonism may actively reduce Aβ(1–42)-mediated increases in cell excitability (Palop et al., 2007; Varga et al., 2014).

## 5 Conclusions

The results of this study demonstrates that hippocampal LTP is intact in aging V-Lep^○b^-/- mice and their wildtype littermates, but LTP in transgenic V-Lep^○b^-/- mice is significantly. This deficit may relate to the significant decreases in hippocampal brain volume that we detected in transgenic mice compared to wild-types and to differences in passive and active neuronal properties that were evident in CA1 pyramidal cells. Aβ(1–42) more potently impaired LTP in the transgenic mice compared to wild-types. Treatment with an amylin receptor antagonist enhanced LTP in both mouse genotypes, and rescued LTP in Aβ(1–42)-treated transgenic mice. In sum, our data indicate that a T2D-like state in rodents results in an increased vulnerability of the hippocampus to the debilitating effects of oligomeric Aβ(1–42) and that effects are derived from changes in metabolic homeostasis related to amylin receptor signaling.

## Data availability statement

The raw data supporting the conclusions of this article will be made available by the authors, upon reasonable request.

## Ethics statement

The animal study was approved by Landesamt für Arbeitsschutz, Naturschutz, Umweltschutz und Verbraucherschutz (LANUV), Arnsberg, Germany. The study was conducted in accordance with the local legislation and institutional requirements.

## Author contributions

MT: Formal analysis, Investigation, Methodology, Writing – review & editing. TH: Formal analysis, Investigation, Methodology, Writing – review & editing. OS: Formal analysis, Investigation, Methodology, Writing – review & editing. JC-K: Investigation, Methodology, Writing – review & editing. AK: Methodology, Writing – review & editing. TB: Formal analysis, Writing – review & editing. DM-V: Conceptualization, Formal analysis, Funding acquisition, Methodology, Resources, Supervision, Writing – original draft, Writing – review & editing.
